# The value of color Doppler ultrasonography combined with serum tumor markers in differential diagnosis of gastric stromal tumor and gastric cancer

**DOI:** 10.1515/med-2023-0805

**Published:** 2023-11-14

**Authors:** Xinyu Cheng, Jianguo Xia, Qi Xu, Huawei Gui

**Affiliations:** Department of Ultrasound Diagnosis, Wuhan Fourth Hospital, Wuhan, 430033, China

**Keywords:** gastric stromal tumor, gastric cancer, color Doppler ultrasonography, carcinoembryonic antigen, carbohydrate antigen 19-9

## Abstract

This study aimed to explore the value of color Doppler ultrasonography combined with carcinoembryonic antigen (CEA) and carbohydrate antigen 19-9 (CA19-9) in differential diagnosis of gastric stromal tumor (GST) and gastric cancer (GC). An analysis of the clinical data of 180 patients with clinically suspected gastric space occupying lesions. According to the postoperative pathological results, 180 suspected gastric space-occupying lesion patients were divided into GST group (*n* = 83) and GC group (*n* = 97). Color Doppler ultrasonography, serum tumor markers CEA and CA19-9 were compared. The research results showed that serum CEA and CA19-9 levels were lower in patients with GST group than those with GC group (both *P* < 0.001). With postoperative pathology as the gold standard, detection rates of GST and GC by combination of color Doppler ultrasound (CDUS), serum CEA, and CA19-9 were higher than those of each index alone (both *P* < 0.001). There was no difference between detection rates of GST and GC by combination of CDUS, serum CEA, and CA19-9 (*P* = 0.058). Color Doppler ultrasonography combined with serum tumor markers CEA and CA19-9 tests has a certain differential diagnostic value for GST and GC, which may provide a reliable reference basis for clinical diagnosis and treatment.

## 1 Introduction

Gastric stromal tumor (GST), which is aggressive and potentially malignant, refers to a group of non-epithelial tumors of the stomach without immunohistochemical features of Schwann cells or ultrastructure of smooth muscle cells [1]. Gastric cancer (GC) is a common malignant tumor of the digestive system [2]. Differentiating between GST and GC is crucial as both conditions have distinct pathological features, different prognoses, and require different treatment approaches. Misdiagnosis or delayed diagnosis of either condition can lead to inadequate treatment, affecting patient outcomes. Moreover, GSTs, although relatively rare, have an increasing incidence and prevalence globally, necessitating greater awareness and understanding of the disease. Differentiation helps to ensure appropriate management and treatment decisions, preventing unnecessary surgical interventions, and improving long-term outcomes. Therefore, understanding the differences between GST and GC is essential for accurate diagnosis, timely treatment, and improving patient outcomes. Furthermore, accurate diagnosis and differentiation between GST and GC are crucial for treatment decision-making and determining patient prognosis. In contrast to GC, surgical resection is generally the first-line treatment option for GST. However, if misdiagnosed or if the tumor regresses or metastasizes, the prognosis can be significantly worse. Therefore, improving understanding of the differences between GST and GC and increasing awareness of the increased incidence of GST may help to improve early detection and appropriate management, ultimately leading to improved patient outcomes.

Both GST and GC feature epigastric discomfort, abdominal pain, upper gastrointestinal bleeding, and abdominal mass, hence the difficulty in their clinical distinguishing. GST and GC are often detected clinically by ultrasound endoscopy or CT and MR enhancement scan, but ultrasound endoscopy is invasive and CT and MR enhancement scan are highly expensive [3,4].

Transabdominal color Doppler ultrasound (CDUS) is a simple imaging tool to differentiate GST from GC, but missed diagnosis may occur [5]. Its efficacy in differential diagnosis of GST remains controversial. Some studies have shown that transabdominal CDUS has a better differential diagnosis of GST [6]. In contrast, it was also found that only 16 lesions were detected by abdominal ultrasound in 88 GST patients. Abdominal ultrasound was believed to be of little value as it can only detect extraluminal, metastatic lesions and a small number of intraluminal lesions [7].

Carcinoembryonic antigen (CEA) and carbohydrate antigen 19-9 (CA19-9) are important serum tumor markers. CEA levels in human serum increase significantly in the presence of tumors, so it is associated with tumor diagnosis and prognosis [8]. CA19-9, in the form of salivary mucin, exists in human serum. It is a mucin-like tumor marker and has demonstrated some value in the diagnosis of tumors [9].

CEA and CA19-9 have been reported to be used in the diagnosis of gastrointestinal cancers, but there are also misdiagnoses and missed diagnoses [10]. The known information is that CEA and CA19-9 are both tumor markers that have been used in the diagnosis and monitoring of cancer, including GC. Color Doppler ultrasonography is a non-invasive imaging technique that can provide detailed information on blood flow in and around tumors. However, the combination of these methods in the differential diagnosis of GST and GC is not well-established.

The purpose of this study was to investigate the value of color Doppler ultrasonography combined with CEA and CA19-9 in differential diagnosis of GST and GC.

## 2 Materials and methods

### 2.1 Patients

A total of 180 patients admitted to our hospital with clinically suspected gastric space occupying lesions from January 2015 to May 2021 were selected as subjects for the study. Inclusion criteria were that patients with suspected gastric space occupying lesions; patients with clinical symptoms such as vague pain and distension in the upper abdomen.

Patients received preoperative transabdominal color Doppler ultrasonography, and the diagnosis of GST or GC in all cases was confirmed by the gold standard of postoperative pathological findings; age ≥18 years; single tumor; complete clinical data; patients and families gave informed consent and voluntarily participated in the study.

Exclusion criteria were that patients combined with other gastric diseases such as gastric polyps and gastritis; patients combined with serious primary cardiovascular and cerebrovascular diseases, liver, kidney abnormalities, etc.; patients combined with blood diseases and infectious diseases; patients who did not cooperate; patients with visual and hearing impairment; patients with GST and GC; patients with other malignant tumors; women during pregnancy and lactation; patients with mental illness; and patients having undergone radiotherapy or chemotherapy before surgery.

### 2.2 Transabdominal CDUS examination method

All patients received preoperative examination by transabdominal CDUS (C5-1 probe, frequency 3.5–5.0 MHz). They were asked to fast for over 8 h before the examination. The patient was placed in supine position, and a whole multi-sectional transabdominal ultrasound scan was performed to observe gastric fundus, body, and antrum. The size was measured and the location determined after detection of the tumor mass. Internal echoes, relationship between the tumor mass and the gastric wall, clarity of the gastric wall stratification, and presence of perigastric lymph node enlargement were closely observed. Distribution of blood flow within and around the tumor mass was observed by color Doppler ultrasonography. If the tumor was in close proximity to the stomach and bladder, the stomach and bladder needed to be filled before the examination. The color Doppler ultrasonography imaging appearance was compared with pathological findings (the gold standard).

### 2.3 Pathological detection method

GST and GC were diagnosed by routine postoperative pathological examination. Specifically, surgically resected specimens were taken from all patients, routinely prepared, HE stained, and observed under light microscopy. Cancers were classified according to the diagnostic criteria from Rosai and Ackerman’s Surgical Pathology [11]. If tumor <3 cm in diameter, no cellular heterogeneity seen, no nuclear division phase; borderline, tumor size of 3–5 cm, dense cells, mild heterogeneity, occasional focal infiltration, and nuclear division phase of 1-4/50 HPFs: the tumor was judged as benign, i.e., GST. If tumor ≥5 cm in diameter, dense cells, marked heterogeneity, infiltrative growth, and nuclear division phase ≥5/50 HPFs, the tumor was judged as malignant, namely GC.

According to the postoperative pathological results, 180 suspected gastric space-occupying lesion patients were divided into GST group (*n* = 83) and GC group (*n* = 97).

### 2.4 Serum tumor markers detection method

Preoperative fasting venous blood was collected from all study subjects in the early morning, and serum was extracted after centrifugation. Levels of serum tumor markers CEA and CA19-9 were measured by electrochemiluminescence. Postoperative pathological results were used as the gold standard to compare the detection rates of GST and GC by color Doppler ultrasonography, serum tumor markers CEA and CA19-9 alone, and the combination of the three.

### 2.5 Statistic analysis

SPSS 25.0 was used for data processing. Measurement data conforming to the normal distribution were represented by mean ± SD, and independent sample *t*-test was used for measurement data comparison. Count data were expressed as *n* (%), and chi-square test was used. *P* ＜ 0.05 indicated that the difference was statistically significant.


**Informed consent:** Informed consent has been obtained from all individuals included in this study.
**Ethical approval:** The research related to human use has been complied with all the relevant national regulations, institutional policies and in accordance with the tenets of the Helsinki Declaration and has been approved by the authors’ institutional review board or equivalent committee.

## 3 Results

### 3.1 Demographic characteristic of the patients

Of the 83 patients with GST group confirmed by postoperative pathology, 54 (65.06%) were male and 29 (34.94%) female, aged 35–72 years, with a mean of (52.49 ± 8.15) years; among the 97 patients with GC group, 60 (61.86%) were male and 37 (38.14%) female, aged 33–73 years, with a mean of (52.37 ± 7.96) years.

### 3.2 Comparison of serum marker levels

The serum CEA and CA19-9 levels were significantly lower in patients with GST group than those with GC group, and the differences were statistically significant (both *P* < 0.001, Table 1).

**Table 1:** Comparison of serum marker levels between GST group and GC group

Parameter	GST group (*n* = 83)	GC group (*n* = 97)	*P* value
Serum CEA (μg/L)	9.10 ± 0.84	13.48 ± 1.39	<0.001
Serum CA19-9 (U/mL)	35.81 ± 3.33	75.63 ± 5.76	<0.001

### 3.3 Preoperative color Doppler ultrasonography and postoperative pathological findings

**Figure 1:**
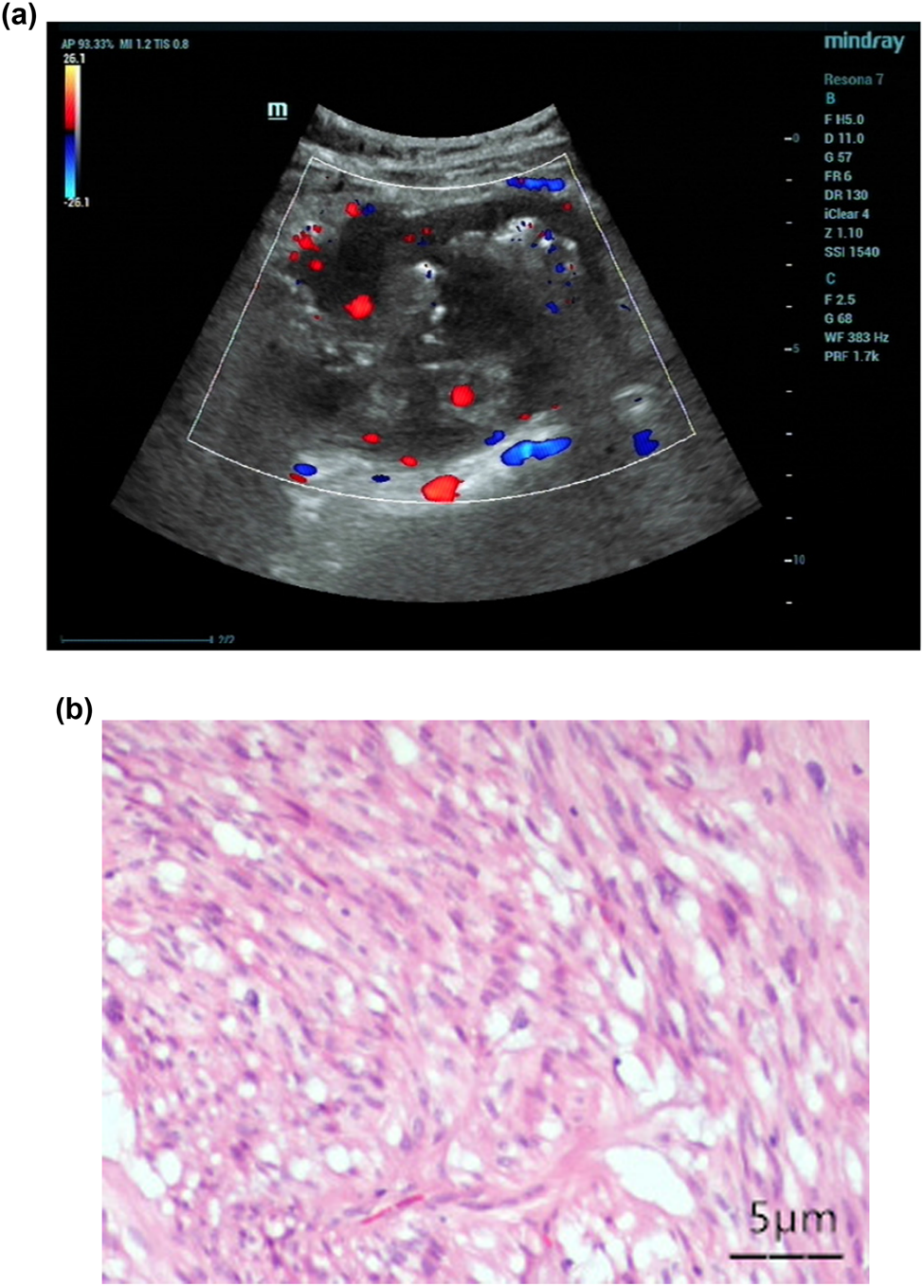
(a) Preoperative color Doppler ultrasonography in a GST patient: solid lesion in the right mid-upper abdomen (below the lower pole of the right kidney and above the right side of the inferior vena cava). (b) Postoperative pathological findings of a GST patient: high-risk gastrointestinal stromal tumor (multiple masses with visible necrosis and vascular invasion).

**Figure 2:**
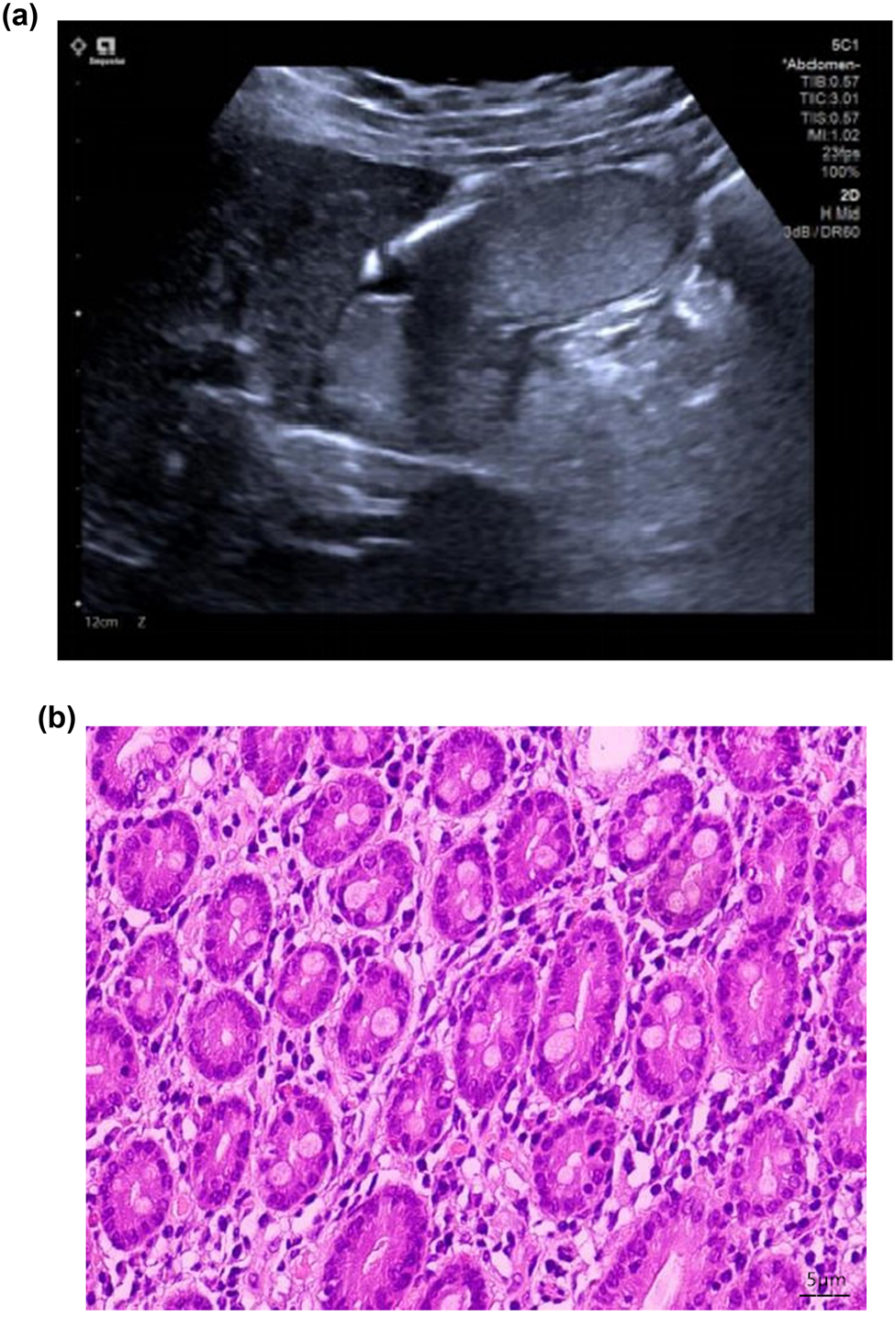
(a) Ultrasonogram of a normal stomach (an ultrasound image of the stomach in a filling state in a healthy person, where the gastric cavity is filled and enlarged, showing liquid echoes with small bubbles floating, and the layered structure of the gastric wall is clearly displayed). (b) Pathological picture of a normal stomach (in a healthy person, the stomach has a normal gastric mucosal layer and gastric glands, and the complete glandular structure can be seen).

CDUS imaging appearance of GST group: round-like and elliptical in shape, mainly found in gastric fundus and body. Ultrasonography showed most masses were well encapsulated and hypoechoic with expansive growth pattern and regular morphology. Liquefaction necrosis appeared in some lesions in which the blood flow was abundant and no lymphadenectasis was found (Figure 1a and b for typical case pictures of the same GST patient; Figure 2a and b for typical pictures of healthy person).

CDUS imaging appearance of GC group: mainly found in gastric antrum, mostly presented as ill-defined margin irregular hypoechoic lesions with invasive growth pattern and pseudokidney sign was observed because of the lesions growing around the gastric cavity. Blood flow was not abundant and lymphadenectasis was found in some lesions.

### 3.4 Comparison of the detection rates of each index alone and in combination for GST and GC

With postoperative pathology as the gold standard, the detection rates of GST by the combination of CDUS, serum CEA, and CA19-9 were significantly higher than those of each index alone, and the difference was statistically significant (*P* < 0.001, Table 2).

**Table 2:** Comparison of the detection rates of each index alone and in combination for GST and GC (*n*[%])

Parameter	CDUS	Serum CEA (cutoff value = 10 μg/L)	Serum CA19-9 (cutoff value = 39 U/mL)	Combined examinations of CDUS, serum CEA, and CA19-9	*P* value
GST group (*n* = 83)	66 (79.52)	15 (18.07)	10 (12.05)	77 (92.77)*	<0.001
GC group (*n* = 97)	70 (72.16)	39 (40.21)	45 (46.39)	81 (83.51)	<0.001

*Note: Compared with the detection rate of combined examinations of CDUS, serum CEA, and CA19-9 for GC, *P* = 0.058.

With postoperative pathology as the gold standard, the detection rates of GC by the combination of CDUS, serum CEA, and CA19-9 were significantly higher than those of each index alone, and the difference was statistically significant (*P* < 0.001, Table 2).

There was no statistically significant difference between the detection rates of GST and GC by the combination of CDUS, serum CEA, and CA19-9 (*P* = 0.058, Table 2).

## 4 Discussion

Distinguishing between GC and GST beforehand by using different combinations can have significant value in deciding the most appropriate treatment plan for the patient post-surgery. While surgical resection is the first-line treatment for both types of cancer, patients diagnosed with GC or GST may require different treatments, such as chemotherapy or radiation therapy, depending on their specific diagnosis. By accurately distinguishing between GC and GST beforehand through the use of multiple combinations, doctors can tailor a treatment plan specific to the patient’s individual needs, improving their chances of successful recovery. Additionally, distinguishing between GC and GST beforehand can help doctors better understand the characteristics and behaviors of each type of cancer, leading to the development of more effective treatment options in the future.

This study found that Color Doppler ultrasonography combined with serum tumor markers CEA and CA19-9 tests has a certain differential diagnostic value for GST and GC. Transabdominal CDUS has been shown to be effective in differential diagnosis of intestinal stromal tumor and intestinal cancer [12]. However, GST and GC each show characteristic presentations on CDUS, and the effectiveness of differential diagnosis between the two remains unclear. Their difference in the origin and growth pattern is the pathological basis for CDUS differentiation.

Originating in the submucosa and growing swollen in the myocardium of the gastric wall, GSTs are mostly convex to the subplasma or submucosa, and they are encapsulated tumors with regular margins at an early stage [13]. CDUS imaging appearance of GST showed characteristics of round-like or elliptical shape, hypoechoic mass with clear margins, and uniform internal echogenicity [14].

GC starts from the mucosal epithelium and grows as infiltrative thickening of the gastric wall [15]. CDUS imaging appearance of GC showed characteristics like ill-defined margin irregular hypoechoic lesions, pseudokidney sign, shallower and wider mucosal ulceration surface compared with stomal tumors [16].

Serum tumor marker detection is convenient, non-invasive, and widely applied in clinical practice. This approach contributes to improving the diagnostic accuracy of tumor patients, which provides a basis for formulating effective treatment plans and evaluating the prognostic quality of patients. However, there is a lack of effective diagnostic markers for GST. One study showed that serum CEA and CA19-9 levels were elevated in GST patients, suggesting that they may be potential markers [17].

The results of this study showed that serum CEA and CA19-9 levels were significantly lower in GST patients compared with patients with GC, suggesting that serum CEA and CA19-9 are of more significant value in the diagnosis of GC. However, it should be noted that in the GC group, clinical features of different patients such as age and tumor, node, metastasis may affect the serum CEA and CA19-9 levels of GC patients. Previous studies also showed that the preoperative serum CA19-9 levels were significantly higher in patients with GC, and the preoperative serum CA19-9 can be detected to identify the staging in patients with GC [18].

The positive rate of CEA and CA19-9 in early GC has been shown to be low in previous studies [19].The detection rates of GST and GC by combined CDUS and serum CEA and CA19-9 were significantly higher than those by each index alone, but the difference was not statistically significant when comparing the detection rates of GST and GC by combining CDUS and serum CEA and CA19-9. It should be noted that there were many similar studies discussing the increased detection rate of the combination of CDUS and serum tumor markers in different tumors [20,21].

The novelty of this study is that it aims to provide evidence of the usefulness of combining color Doppler ultrasonography with CEA and CA19-9 in the differential diagnosis of GST and GC. This study will contribute to the development of a more accurate, non-invasive, and cost-effective diagnostic approach for these two conditions.

This study did not design a normal control group, which is a limitation. The author did not perform receiver operator characteristic (ROC) analysis in this study due to limited sample size. The author will take into consideration the possibility of performing ROC analysis in future studies with a larger sample size. The relationship between GST and GC is important to consider when analyzing the diagnostic accuracy of a tool used to distinguish between the two conditions. If GST and GC are simply different stages of the same disease process, it could impact the interpretation of the results. For example, a tool that accurately diagnoses GC may also diagnose GST, but may not accurately differentiate between the two. This could lead to false positives or negatives, affecting the tool’s sensitivity and specificity. In addition, the 180 patients seem to be diagnosed to have either GST or GC. Many of other patients are excluded. For next research the author needs to have some people without either of the two conditions to test sensitivity and specificity.

## 5 Conclusion

Color Doppler ultrasonography combined with serum tumor markers CEA and CA19-9 tests has a certain differential diagnostic value for GST and GC, which can provide a reliable reference basis for clinical diagnosis and treatment.
